# Predicting the purebred-crossbred genetic correlation from the genetic variance components in the parental lines

**DOI:** 10.1186/s12711-021-00601-w

**Published:** 2021-02-04

**Authors:** Pascal Duenk, Piter Bijma, Yvonne C. J. Wientjes, Mario P. L. Calus

**Affiliations:** Animal Breeding and Genomics, Wageningen University and Research, P.O. Box 338, 6700 AH Wageningen, The Netherlands

## Abstract

**Background:**

The genetic correlation between purebred and crossbred performance (${r}_{pc}$) is an important parameter in pig and poultry breeding, because response to selection in crossbred performance depends on the value of ${r}_{pc}$ when selection is based on purebred (PB) performance. The value of ${r}_{pc}$ can be substantially lower than 1, which is partly due to differences in allele frequencies between parental lines when non-additive genetic effects are present. This relationship between ${r}_{pc}$ and parental allele frequencies suggests that ${r}_{pc}$ can be expressed as a function of genetic parameters for the trait in the parental lines. In this study, we derived expressions for ${r}_{pc}$ based on genetic variances within, and the genetic covariance between parental lines. It is important to note that the variance components used in our expressions are not the components that are typically estimated in empirical data. The expressions were derived for a genetic model with additive and dominance effects (D), and additive and epistatic additive-by-additive effects (E_AA_). We validated our expressions using simulations of purebred parental lines and their crosses, where the parental lines were either selected or not. Finally, using these simulations, we investigated the value of ${r}_{pc}$ for genetic models with both dominance and epistasis or with other types of epistasis, for which expressions could not be derived.

**Results:**

Our simulations show that when non-additive effects are present, ${r}_{pc}$ decreases with increasing differences in allele frequencies between the parental lines. Genetic models that involve dominance result in lower values of ${r}_{pc}$ than genetic models that involve epistasis only. Using information of parental lines only, our expressions provide exact estimates of ${r}_{pc}$ for models D and E_AA_, and accurate upper and lower bounds of ${r}_{pc}$ for two other genetic models.

**Conclusion:**

This work lays the foundation to enable estimation of ${r}_{pc}$ from information collected in PB parental lines only.

## Background

Pig and poultry breeders benefit from heterosis and breed complementarity by mating animals from genetically-distinct purebred parental lines to produce crossbred production animals [1, 2]. The aim of such breeding programs is to improve crossbred (CB) performance, while selection is within the parental lines, usually based on measurements of purebred (PB) performance. As a result, response to selection in CB performance depends partly on the genetic correlation between PB and CB performance (${r}_{pc}$), which is generally lower than 1 for most traits in livestock populations [3–9]. Hence, ${r}_{pc}$ is an important parameter in breeding programs of pig and poultry.

Estimates of ${r}_{pc}$ can be obtained with models that use phenotypic information on both PB and CB performance. Such models require either pedigree information that links the CB to the PB animals [4, 10], or genotype information on both PB and CB animals [11]. Tracking pedigree in a crossbreeding system is often impractical, and collecting phenotypes and genotypes on CB animals may be difficult and costly. Furthermore, breeding companies may produce many different crosses between parental lines, which makes the effort of estimating all relevant ${r}_{pc}$ even more costly. To overcome these issues, it would be beneficial if ${r}_{pc}$ could be estimated based on data from the parental PB lines, instead of requiring CB data.

The ${r}_{pc}$ can be lower than 1 due to (1) differences in trait definition between PB and CB performance [12, 13], (2) genotype-by-environment interactions (G $\times$ E) [10, 14], and (3) genotype-by-genotype interactions (G $\times$ G) in combination with differences in allele frequencies between the parental lines at loci that affect the trait [9, 15, 16], i.e. the quantitative trait loci (QTL). G $\times$ G interactions result from non-additive genetic effects (i.e. dominance and epistasis). Here, we consider only the impact of G $\times$ G interactions on ${r}_{pc}$, assuming that there are no G $\times$ E interactions. The impact of non-additive effects on ${r}_{pc}$ has been studied by Wei et al*.* [15] and Baumung et al*.* [16], who derived expressions for ${r}_{pc}$ in terms of known additive, dominance, and epistatic genetic effects of loci, and as a function of differences in allele frequencies at these loci between the parental lines. These expressions were, however, limited to one- and two-locus models, and thus cannot be used to predict ${r}_{pc}$ for traits that are highly polygenic. Furthermore, genetic effects and allele frequencies at the QTL are usually unknown. Thus, for polygenic traits, there is a need for expressions of ${r}_{pc}$ that are based on observable parameters in the parental lines.

Previously, we investigated the impact of non-additive effects on the additive genetic correlation (${r}_{g}$) for a trait between breeding lines [17] and showed that ${r}_{g}$ decreases with increasing size of non-additive effects, and with increasing differences in allele frequencies at QTL between the lines. In the current study, we investigate the impact of non-additive effects on the relationship between ${r}_{g}$ and ${r}_{pc}$.

While estimation of ${r}_{g}$ between two PB lines is relatively straightforward with a genomic relationship matrix [18], the interpretation of the resulting estimates requires careful consideration. Following Duenk et al*.* [17], we define ${r}_{g}$ between line 1 and line 2 as the correlation between additive genetic values of the individuals in line 1, for the trait expressed in lines 1 and 2. In other words, suppose we know the average effects of all QTL in lines 1 and 2, then we can calculate two additive genetic values for the individuals in line 1; one based on the average effects in line 1, and one based on the average effects in line 2. The ${r}_{g}$ between lines 1 and 2 for line 1 is the correlation between these two additive genetic values:

1 $${r}_{g}=\frac{{\sigma }_{\mathrm{1,1}\left(2\right)}}{\sqrt{{\sigma }_{1}^{2}}\sqrt{{\sigma }_{1\left(2\right)}^{2}}}$$

In Eq. (1), ${\sigma }_{1}^{2}$ is the ordinary additive genetic variance for PB performance in line 1; ${\sigma }_{1\left(2\right)}^{2}$ is the additive genetic variance in line 1 for the trait expressed in line 2, which depends on the allele frequencies in line 1 and the average effects for performance in line 2; and, similarly, ${\sigma }_{\mathrm{1,1}\left(2\right)}$ is the additive genetic covariance in line 1 between the trait expressed in line 1 and the trait expressed in line 2, which depends on allele frequencies in line 1 and the average effects for the trait in lines 1 and 2. Note that ${\sigma }_{1(2)}^{2}$ differs from the ordinary additive genetic variance for purebred performance in line 2 (i.e. ${\sigma }_{2}^{2}$). Similarly, the covariance also differs between lines 1 and 2, i.e. ${\sigma }_{\mathrm{1,1}\left(2\right)}\ne {\sigma }_{\mathrm{2,2}\left(1\right)}$; while both covariances depend on the average effects in both lines, ${\sigma }_{\mathrm{1,1}\left(2\right)}$ depends on the allele frequencies in line 1 and ${\sigma }_{\mathrm{2,2}\left(1\right)}$ depends on the allele frequencies in line 2. Therefore, ${r}_{g}$ for line 2 is a different parameter because it depends on allele frequencies in line 2. Here, we focus on ${r}_{g}$ for line 1, because we are interested in ${r}_{pc}$ for line 1.

Our aim was to derive expressions for the prediction of ${r}_{pc}$ in a two-way crossbred breeding program, based on genetic variances within the parental lines and the genetic covariance between the parental lines (i.e., the terms in Eq. (1)). The resulting expressions predict the component of ${r}_{pc}$ that is due to non-additive effects. Expressions were derived for two genetic models; a genetic model with additive and dominance effects (D), and a genetic model with additive and additive-by-additive (A $\times$ A) epistatic effects between pairs of QTL (E_AA_). We validated our expressions using simulations of PB parental lines and their crosses, where the parental lines were either selected or not. Finally, using simulations, we also investigated the value of ${r}_{pc}$ for two genetic models for which expressions could not be derived: a model with both dominance and A $\times$ A epistatic effects (D + E_AA_) and a model with complementary epistatic effects (E_C_). We compared the results from these models with our predictions of ${r}_{pc}$ under models D and E_AA_.

## Theory

We consider two PB parental lines (1 and 2) that are mated to produce CB individuals. The additive genetic correlation between PB and CB performance (${r}_{pc}$) in line 1 is defined as the correlation between additive genetic values for PB and CB performance of members of line 1 [15, 19]. For PB performance, the additive genetic value of individual $i$ from line 1 is:

2 $${v}_{i,1}={\mathbf{h}}_{i}^{\mathrm{^{\prime}}}{{\varvec{\upalpha}}}_{1}$$

where ${\mathbf{h}}_{i}$ is a column vector of genotypes of individual $i$ at all QTL (measured as allele counts minus the average allele count in line 1), and ${{\varvec{\upalpha}}}_{1}$ is a column vector of average effects of allele substitution for PB performance at QTL in line 1. Similarly, the additive genetic value of individual $i$ for CB performance is:

3 $${v}_{i,C}={\mathbf{h}}_{i}^{\mathrm{^{\prime}}}{{\varvec{\upalpha}}}_{1(C)}$$

where ${{\varvec{\upalpha}}}_{1(C)}$ is a column vector of average effects of allele substitution for CB performance at QTL in line 1. Here and throughout the remainder of this paper, genotypes (${\mathbf{h}}_{i}$) are considered random variables, whereas the average effects (${{\varvec{\upalpha}}}_{1}$, ${{\varvec{\upalpha}}}_{1(C)}$) are considered fixed.

The ${r}_{pc}$ in line 1 is the correlation between the additive genetic values in Eqs. (2) and (3):

4 $${r}_{pc}=cor\left({v}_{i,1},{v}_{i,C}\right)=\frac{cov\left({v}_{i,1},{v}_{i,C}\right)}{\sqrt{var\left({v}_{i,1}\right)}\sqrt{var\left({v}_{i,C}\right)}}=\frac{{\sigma }_{\mathrm{1,1}\left(C\right)}}{{\sigma }_{1}{\sigma }_{1\left(C\right)}}$$

where ${\sigma }_{1}$ is the additive genetic standard deviation for PB performance in line 1, ${\sigma }_{1\left(C\right)}$ is the additive genetic standard deviation for CB performance in line 1, and ${\sigma }_{\mathrm{1,1}(C)}$ is the additive genetic covariance between PB and CB performance in line 1.

Our aim is to express ${r}_{pc}$ in terms of genetic parameters in the parental lines. First, we derive expressions for ${\alpha }_{1}$ and ${\alpha }_{1(C)}$ for a genetic model with additive and dominance effects (D), and for a model with additive and additive-by-additive epistatic effects (E_AA_). Second, we express ${\alpha }_{1(C)}$ in terms of average effects of allele substitution for PB performance in the parental lines (${\alpha }_{1}$ and ${\alpha }_{2}$). Third, we derive expressions for ${\sigma }_{\mathrm{1,1}(C)}$ and ${\sigma }_{1(C)}$, and finally for ${r}_{pc}$, in terms of genetic parameters in the parental lines.

### Derivation of average effects of allele substitution for PB and CB performance

The first step is to derive expressions for average effects of allele substitution in line 1 for PB and CB performance. For CB performance, we are interested in the effect of alleles from line 1 on genotypic values of CB offspring (i.e., when line 1 is randomly mated to line 2). Hence, we want to express the average effects of allele substitution in terms of differences between genotypic values of CB offspring. Following Falconer [20], the average effect of an allele is the mean genotypic value of offspring produced by transmitting that allele, minus the mean genotypic value of the population. The average effect of allele substitution at a bi-allelic locus is equal to the difference between the average effects of its alleles. Strictly speaking, this is the definition of average excess, but it is equivalent to the average effect under random mating [20]. Hence, if individuals of line 1 are mated at random to individuals of line 2, then the average effect and average excess are identical, even though the resulting CB population is not in Hardy–Weinberg equilibrium. In the following, we will use the term ‘average effect’ to refer to the average effect of allele substitution at a locus. Furthermore, we assume that the genetic additive, dominance, and epistatic effects are the same for PB and CB performance, and that for CB performance, these effects are independent of line origin. Statistical additive, dominance and epistatic effects, however, are line-dependent due to differences in allele frequencies. In other words, ${r}_{pc}$ values lower than one are the result of G $\times$ G interaction.

#### Dominance model (D)

Consider a locus that has an additive effect ($a$), a dominance effect ($d$), and no epistatic interactions with other loci. The average effect for PB performance in line 1 under this genetic model (D) is equal to:

5 $${\alpha }_{1}^{D}=a+\left(1-2{p}_{1}\right)d$$

where ${p}_{1}$ is the frequency of the focal allele in line 1. The full derivation leading to this result can be found in Falconer and Mackay [19], and the average effect for CB performance in line 1 when mated to line 2 can be derived in a similar way. In contrast to alleles transmitted to PB animals, alleles from line 1 transmitted to crossbreds will always pair with an allele from line 2. Thus, the average effect for CB performance in line 1 under genetic model D depends on the allele frequency in line 2 only [21, 22]:

6 $${\alpha }_{1(C)}^{D}=a+\left(1-2{p}_{2}\right)d$$

where ${p}_{2}$ is the frequency of the focal allele in line 2. Thus, under genetic model D, the average effect for CB performance in line 1 when mated to line 2 is equal to the average effect for PB performance in line 2 (${\alpha }_{2}^{D}$) (see also Zeng et al*.* [23] and Vitezica et al*.* [24]).

#### Additive-by-additive epistasis model (E_AA_)

With additive-by-additive (A $\times$ A) epistasis (i.e. genetic model E_AA_), and without dominance or other types of epistasis, the average effect at a locus does not depend on the allele frequency at the focal locus, but on the allele frequency at the loci it interacts with. Consider a locus F with alleles $F$ and $f$, which has an additive effect ($a$), and an A $\times$ A epistatic interaction with locus G with alleles $G$ and $g$. The epistatic effect between F and G is denoted as $\epsilon$. For simplicity of presentation, we assume in the following derivation that locus G has no additive effect, because the result for locus F does not depend on the additive effect at locus G. In addition, we only considered pairwise interactions between loci, as opposed to interactions between more than two loci. Table 1 shows the genotypic values for the two-locus genotypes, e.g. [25]. The genotypic values are the sum of the additive effect at locus F, and the epistatic effect between loci F and G. The sign in front of the additive effect depends on the genotype at locus F, whereas the sign in front of the epistatic effect depends on the genotype at both F and G.

**Table 1:** Genotypic values of two locus (F and G) genotypes with additive-by-additive (A $\times$ A) epistasis (model E_AA_)

Frequency		${P}^{\mathrm{F}}$	${H}^{\mathrm{F}}$	${Q}^{\mathrm{F}}$
		$FF$	$Ff$	$ff$
${P}^{\mathrm{G}}$	$GG$	$a+\epsilon$	0	$-a-\epsilon$
${H}^{\mathrm{G}}$	$Gg$	$a$	0	$-a$
${Q}^{\mathrm{G}}$	$gg$	$a-\epsilon$	0	$-a+\epsilon$

The genotypic values are the sum of the additive effect at locus F, and the epistatic effect between loci F and G. The sign in front of the additive effect depends on the genotype at locus F, whereas the sign in front of the epistatic effect depends on the genotype at loci F and G. For simplicity, it is assumed that G has no additive effect

${P}^{X}$, ${H}^{X}$*,* and ${Q}^{X}$ denote the genotype frequencies at locus X, $a$ denotes the additive effect at locus F, and $\epsilon$ denotes the epistatic effect between loci F and G

The average effect at locus F for PB performance in line 1 can be derived by computing the difference between the average effects of alleles $F$ and $f$. The average effect of an allele for PB performance of the same line is the mean genotypic value of offspring that inherited that allele from line 1, assuming the other allele was drawn at random from line 1. The average effect of allele $F$ in line 1 is:

$${\alpha }^{F}={p}_{1}^{F}\left({P}_{1}^{G}\left(a+\epsilon \right)+{H}_{1}^{G}\left(a\right)+{Q}_{1}^{G}\left(a-\epsilon \right)\right)+ {p}_{1}^{f}\left({P}_{1}^{G}\left(0\right)+{H}_{1}^{G}\left(0\right)+{Q}_{1}^{G}\left(0\right)\right)={p}_{1}^{F}\left(\left({P}_{1}^{G}-{Q}_{1}^{G}\right)\epsilon +a\right)$$

where ${P}_{1}^{G}$, ${H}_{1}^{G}$, and ${Q}_{1}^{G}$ are genotype frequencies at locus G in line 1 (Table 1), ${p}_{1}^{F}$ is the frequency of allele $F$ in line 1, and ${p}_{1}^{f}=1-{p}_{1}^{F}$. Similarly, the average effect of allele $f$ is:

$${\alpha }^{f}={p}_{1}^{F}\left({P}_{1}^{G}\left(0\right)+{H}_{1}^{G}\left(0\right)+{Q}_{1}^{G}\left(0\right)\right)+{p}_{1}^{f}\left({P}_{1}^{G}\left(-a-\epsilon \right)+{H}_{1}^{G}\left(-a\right)+{Q}_{1}^{G}\left(a+\epsilon \right)\right)={-p}_{1}^{f}\left(\left({P}_{1}^{G}-{Q}_{1}^{G}\right)\epsilon +a\right)$$

The average effect at locus F for PB performance in line 1 under genetic model E_AA_ then is:

7 $$\alpha_{1}^{AA} = \alpha^{F} - \alpha^{f} = (p_{1}^{F} + p_{1}^{f} )((P_{1}^{G} - Q_{1}^{G} )\epsilon + a) = a + (P_{1}^{G} - Q_{1}^{G} )\epsilon = a - \left( {1 - 2p_{1}^{G} } \right)\epsilon$$

where ${p}_{1}^{G}$ is the frequency of allele $G$ in line 1. The average effect for PB performance in line 2 can be obtained by using the allele frequency in line 2 in Eq. (7).

The average effect for CB performance in line 1 can be derived similarly using the expected genotype frequencies in CB offspring at locus G. This results in:

8 $${\alpha }_{1(C)}^{AA}=a-\left(1-2{p}_{C}^{G}\right)\epsilon$$

where ${p}_{C}^{G}$ is the expected frequency of allele $G$ in the CB offspring. Given the expressions for ${\alpha }_{1}^{AA}$ and ${\alpha }_{2}^{AA}$ (Eqs. (7) and (8)), and using ${p}_{C}^{G}=0.5\left({p}_{1}^{G}+{p}_{2}^{G}\right)$, the average effect of CB performance in line 1 under genetic model E_AA_ can be written as the mean of average effects for PB performance in lines 1 and 2:

9 $${\alpha }_{1(C)}^{AA}=0.5\left({\alpha }_{1}+{\alpha }_{2}\right)$$

### Derivation of ${\mathbf{r}}_{\mathbf{p}\mathbf{c}}$

In the following, we use our derivations of ${\alpha }_{1}$ and ${\alpha }_{1(C)}$ for genetic models D and E_AA_ to derive the additive genetic variance for PB and CB performance in line 1 (${\sigma }_{1}^{2}$ and ${\sigma }_{1\left(C\right)}^{2}$), and the additive genetic covariance between PB and CB performance in line 1 (${\sigma }_{\mathrm{1,1}(C)}$). Then, we use these derivations in an expression for ${r}_{pc}$. In these derivations, we treat the genotypes of individuals as random. We assume that there is no correlation between average effects at different loci, and that the average effect at a locus is independent from the genotypes at that locus as a result of random allele coding (i.e., which allele is the focal allele is independent of the effect of the allele). Regardless of the genetic model, we define the additive genetic variance for PB performance in line 1 as:

10 $$\sigma _{1}^{2} ~ = var\left( {{\mathbf{h}}_{i}^{'} {\mathbf{\alpha }}_{1} } \right) = var\left( {\mathop \sum \limits_{j} h_{{ij}} \alpha _{{1j}} } \right) = E\left[ {\left( {\mathop \sum \limits_{j} h_{{ij}} \alpha _{{1j}} } \right)\left( {\mathop \sum \limits_{j} h_{{ij}} \alpha _{{1j}} } \right)} \right] = \mathop \sum \limits_{j} E\left( {h_{{ij}} h_{{ij}} } \right)\alpha _{{1j}} \alpha _{{1j}} = ~\mathop \sum \limits_{j} 2p_{{1j}} \left( {1 - p_{{1j}} } \right)\alpha _{{1j}}^{2}$$

where $j$ denotes the locus.

#### Dominance model (D)

With dominance (model D), average effects for CB performance in line 1 are equal to average effects for PB performance in line 2 (Eq. (6)). Hence, with model D, the additive genetic variance for CB performance in line 1 is:

11 $$\begin{aligned}{\sigma }_{1(C)}^{2} &=var\left({\mathbf{h}}_{i}^{\mathrm{^{\prime}}}{{\varvec{\upalpha}}}_{1(C)}\right)=var\left(\sum_{j}{h}_{ij}{\alpha }_{2j}\right)\\ &=E\left[\left(\sum_{j}{h}_{ij}{\alpha }_{2j}\right)\left(\sum_{j}{h}_{ij}{\alpha }_{2j}\right)\right]\\ &=\sum_{j}E\left({h}_{ij}{h}_{ij}\right){\alpha }_{2j}{\alpha }_{2j}\\ &= \sum_{j}2{p}_{1j}\left(1-{p}_{1j}\right){\alpha }_{2j}^{2}={\sigma }_{1(2)}^{2}\end{aligned}$$

where ${\sigma }_{{\alpha }_{2}}^{2}$ is the variance of average effects for PB performance in line 2. As mentioned in the Background section, it is important to note that ${\sigma }_{1(2)}^{2}$ is the additive genetic variance in line 1 for the trait expressed in line 2. This is evident from Eq. (11), where $\sum_{j}\left(2{p}_{1j}\left(1-{p}_{1j}\right)\right)$ contains allele frequencies in line 1 and ${\alpha }_{2j}$ is the average effect at locus $j$ for the trait expressed in line 2 (see also Background and Discussion).

The additive genetic covariance between PB and CB performance in line 1 can be written as:

12 $$\sigma _{{1,1\left( C \right)}} = cov\left( {\mathbf{h}}_{i}^{'} {\varvec{\upalpha}}_{1} ,{\mathbf{h}}_{i}^{\prime} {{\varvec{\upalpha}}}_{{1\left( C \right)}} \right) = cov\left( {\mathop \sum \limits_{j} h_{{ij}} \alpha _{{1j}} ,\mathop \sum \limits_{j} h_{{ij}} \alpha _{{2j}} } \right) = E\left[ {\left( {\mathop \sum \limits_{j} h_{{ij}} \alpha _{{1j}} } \right)\left( {\mathop \sum \limits_{j} h_{{ij}} \alpha _{{2j}} } \right)} \right] = \mathop \sum \limits_{j} E\left( {h_{{ij}} h_{{ij}} } \right)\alpha _{{1j}} \alpha _{{2j}} = \mathop \sum \limits_{j} 2p_{{1j}} \left( {1 - p_{{1j}} } \right)\alpha _{{1j}} \alpha _{{2j}} = \sigma _{{1,1\left( 2 \right)}} ,$$

where ${\sigma }_{\mathrm{1,1}(2)}$ is the additive genetic covariance for individuals in line 1 between the trait expressed in lines 1 and 2.

As a result, with model D, ${r}_{pc}$ for line 1 can be written as:

13 $${r}_{pc}^{D}=cor\left({\mathbf{h}}_{i}^{\mathrm{^{\prime}}}{{\varvec{\upalpha}}}_{1},{\mathbf{h}}_{i}^{\mathrm{^{\prime}}}{{\varvec{\upalpha}}}_{2}\right)=\frac{{\sigma }_{\mathrm{1,1}(2)}}{{\sigma }_{1}{\sigma }_{1(2)}}$$

Hence, for model D, ${r}_{pc}$ for line 1 is equal to the correlation between additive genetic values of individuals in line 1 for the trait expressed in parental lines 1 and 2, which is equal to the genetic correlation between lines 1 and 2, as defined in the Background section (Eq. (1)). It is important to note, however, that this is different from the correlation between average effects for PB performance in the parental lines (e.g., Xiang et al*.* [26]), because Eq. (1) is a weighted correlation between average effects, where weights are computed based on genotype frequencies in line 1.

#### Additive-by-additive epistasis (model E_AA_)

With A $\times$ A epistatic interactions (model E_AA_), the average effect for CB performance in line 1 is equal to the mean of the average effects for PB performance in the two parental lines (Eq. (9)). Thus, the additive genetic variance for CB performance in line 1 can be written as:

14 $$\begin{aligned}{\sigma }_{1\left(C\right)}^{2}&=var\left({\mathbf{h}}_{i}^{\mathrm{^{\prime}}}{{\varvec{\upalpha}}}_{1\left(C\right)}\right)=var\left(\sum_{j}{h}_{ij}0.5\left({\alpha }_{1j}+{\alpha }_{2j}\right)\right)\\ &=E\left[\left(\sum_{j}{h}_{ij}0.5\left({\alpha }_{1j}+{\alpha }_{2j}\right)\right)\left(\sum_{j}{h}_{ij}0.5\left({\alpha }_{1j}+{\alpha }_{2j}\right)\right)\right]\\ &=\sum_{j}E\left({h}_{ij}{h}_{ij}\right){\left(0.5\left({\alpha }_{1j}+{\alpha }_{2j}\right)\right)}^{2}\\ &= \, 0.25\sum_{j}2{p}_{1j}\left(1-{p}_{1j}\right)\left({\alpha }_{1j}^{2}+{\alpha }_{2j}^{2}+2{\alpha }_{1j}{\alpha }_{2j}\right)\\ &= 0.25({\sigma }_{1}^{2}+{\sigma }_{1(2)}^{2}+ \, 2{\sigma }_{\mathrm{1,1}(2)})\end{aligned}$$

The additive genetic covariance between PB performance and CB performance in line 1 can be derived as:

15 $$\begin{aligned}{\sigma }_{\mathrm{1,1}\left(C\right)}&=cov\left({\mathbf{h}}_{i}^{\mathrm{^{\prime}}}{{\varvec{\upalpha}}}_{1},{\mathbf{h}}_{i}^{\mathrm{^{\prime}}}{{\varvec{\upalpha}}}_{1\left(C\right)}\right)\\ &=cov\left(\sum_{j}{h}_{ij}{\alpha }_{1j},{h}_{ij}0.5\left({\alpha }_{1j}+{\alpha }_{2j}\right)\right)\\ &=E\left[\left(\sum_{j}{h}_{ij}{\alpha }_{1j}\right)\left(\sum_{j}{h}_{ij}0.5\left({\alpha }_{1j}+{\alpha }_{2j}\right)\right)\right]\\ &=\sum_{j}E\left({h}_{ij}{h}_{ij}\right){\alpha }_{1j}0.5\left({\alpha }_{1j}+{\alpha }_{2j}\right)\\ &=0.5\sum_{j}2{p}_{1j}\left(1-{p}_{1j}\right)\left({\alpha }_{1j}^{2}+{\alpha }_{1j}{\alpha }_{2j}\right)\\ &=0.5{\sigma }_{1}^{2}+0.5{\sigma }_{\mathrm{1,1}(2)}\end{aligned}$$

Hence, ${r}_{pc}$ for line 1 with genetic model E_AA_ is equal to:

16 $$\begin{aligned}{r}_{pc}^{AA}&=\frac{0.5{\sigma }_{1}^{2}+0.5{\sigma }_{\mathrm{1,1}(2)}}{{\sigma }_{1} \sqrt{0.25\left({\sigma }_{1}^{2}+{\sigma }_{1(2)}^{2}+2{\sigma }_{\mathrm{1,1}(2)}\right)}}\\ &=\frac{{\sigma }_{1}^{2}+{\sigma }_{\mathrm{1,1}(2)}}{{\sigma }_{1} \sqrt{\left({\sigma }_{1}^{2}+{\sigma }_{1(2)}^{2}+2{\sigma }_{\mathrm{1,1}(2)}\right)}}\end{aligned}$$

Thus, for genetic model E_AA_, ${r}_{pc}$ is a function of the additive genetic covariance in line 1 between the trait expressed in line 1 and line 2 (${\sigma }_{\mathrm{1,1}(2)}$), and the additive genetic variances in line 1 for the trait expressed in line 1 (${\sigma }_{1}^{2}$) and in line 2 (${\sigma }_{1(2)}^{2}$). The expressions in Eqs. (13) and (16) show that the value of ${r}_{pc}$ due to G $\times$ G interactions can be determined based on genetic parameters within the parental lines.

### Expressions as bounds of ${{\varvec{r}}}_{{\varvec{p}}{\varvec{c}}}$

As evident from our derivations (e.g. Equations (2) and (3)), ${r}_{pc}$ depends on the difference between average effects at QTL for PB and CB performance (${\Delta }_{\alpha }$). With model D, it follows from the difference between Eqs. (5) and (6) that ${\Delta }_{\alpha }$ increases by $2({p}_{1}-{p}_{2})$ per unit increase in the magnitude of the dominance effect. This is because with model D, ${\alpha }_{1(C)}$ at a locus depends on the allele frequency in the mated line, whereas ${\alpha }_{1}$ depends on the allele frequency in line 1. With model E_AA_, in contrast, ${\Delta }_{\alpha }$ increases by $2\left({p}_{1}-{p}_{C}\right)=$ (${p}_{1}-{p}_{2}$) per unit increase in the epistatic effect, based on the difference between Eqs. (7) and (8). This is because with model E_AA_, ${\alpha }_{1(C)}$ of a locus depends on the allele frequency of the interacting locus in the cross, rather than in the mated line. Hence, for each unit increase in the magnitude of non-additive effects, ${\Delta }_{\alpha }$ increases twice as fast with genetic model D as with model E_AA_.

Because any non-additive interaction involves either dominance, epistasis, or both, models D and E_AA_ may represent extremes, where ${r}_{pc}$ either depends on (1) the difference in allele frequency between the parental lines (model D), or (2) half of that difference (model E_AA_). With other genetic models, ${r}_{pc}$ may depend on (1), (2), or both. However, it is unlikely that other genetic models will lead to much lower ${r}_{pc}$ than model D, because the maximum ${\Delta }_{\alpha }$ is bounded by the difference in allele frequencies between parental lines. In addition, it is unlikely that other genetic models will lead to higher ${r}_{pc}$ than predicted with model E_AA_, because the minimum ${\Delta }_{\alpha }$ is bounded by the difference in allele frequencies between line 1 and the cross. Hence, we can expect that ${r}_{pc}$ lies somewhere in between ${r}_{pc}^{D}$ and ${r}_{pc}^{AA}$ for other non-additive genetic models. To tests this hypothesis, we used simulation to evaluate ${r}_{pc}$ for two other non-additive genetic models, as described in the following.

## Methods

**Fig. 1:**
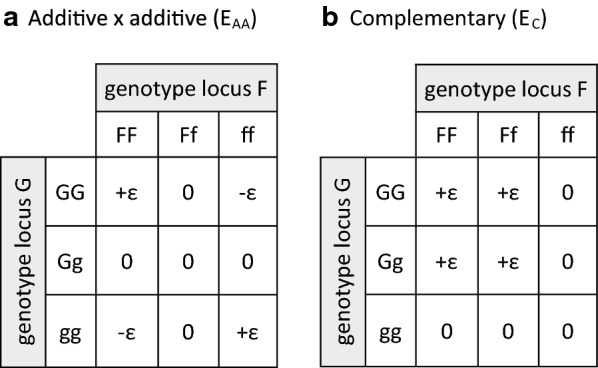
Epistatic contrasts for two functional epistatic configurations. $\epsilon$ is the epistatic effect between loci F and G

Simulation was used to validate the derived expressions for ${r}_{pc}$ (i.e. Equations (13) and (16)). For that purpose, we simulated seven purebred lines that were either positively (P), negatively (N), or randomly selected (R) for the trait of interest. Both positive and negative selection were considered, such that pairs of lines were either selected in the same direction (convergent) or in opposite directions (divergent), resulting in pairs of lines with small and large differences in allele frequencies at QTL. We considered four scenarios that differed in the type of non-additive effects simulated (Fig. 1); (1) only dominance (D), (2) only additive-by-additive (A $\times$ A) epistasis (E_AA_), (3) both dominance and A $\times$ A epistasis (D + E_AA_), and (4) complementary epistasis (E_C_). The latter was chosen because it is expected to result in substantial non-additive variance of all types (i.e. dominance, additive-by-additive, dominance-by-additive, additive-by-dominance, and dominance-by-dominance) [27]. For each scenario and each pairwise cross between parental lines, we computed the realized (i.e. true) ${r}_{pc}$ and compared it with the predicted ${r}_{pc}$ based on Eqs. (13) and (16).

### Simulation

#### Population

**Fig. 2:**
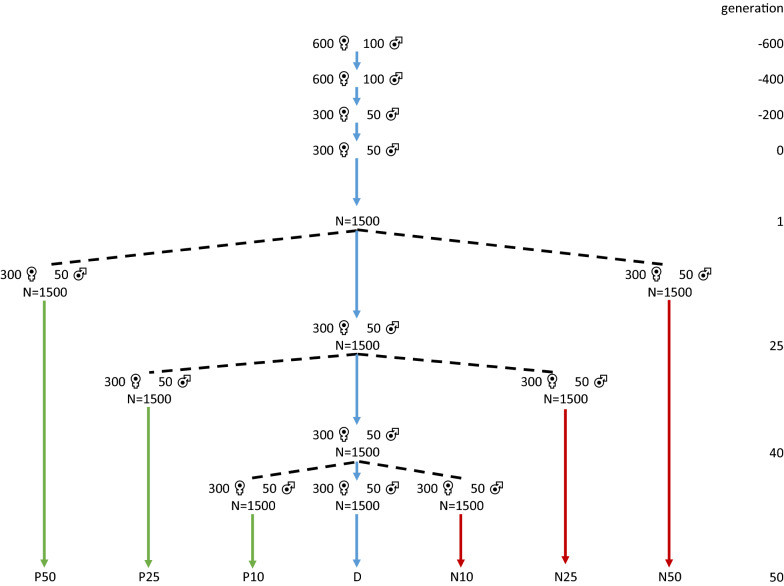
Overview of the simulation of seven breeding lines. Green lines indicate positive selection based on own performance records and red lines indicate negative selection based on own performance. Blue lines indicate random selection

We simulated QTL genotypes of animals from seven breeding lines that originated from a common historical population using QMSim [28], such that the number of generations that separated pairs of lines ranged from 10 to 100 (Fig. 2). First, a historical population was simulated by randomly mating 600 females with 100 males for 200 generations. During the following 200 generations, the population size was gradually decreased to 300 females and 50 males, to generate linkage disequilibrium (LD). Then, mating continued with a constant population size for another 200 generations. In the last historical generation (generation 0), the population size was increased to 1500 males and 1500 females by creating litters of 10 offspring per mating. The effective population size (${N}_{e}$) between generations − 600 and 0 was ~ 234, calculated as the harmonic mean of $\frac{4{N}_{m}{N}_{f}}{{N}_{m}+{N}_{f}}$ in each historical generation, where ${N}_{m}$ is the number of males and ${N}_{f}$ is the number of females that become parents in a generation [19].

From the last historical generation, three breeding lines (P50, R and N50) were created by sampling 300 females and 50 males for each of the lines, without replacement. Within each line, mating continued for 50 generations, by selecting 50 males and 300 females in each generation using truncation selection based on own performance records with a broad sense heritability of 0.3. In line P50, selection was for high performance (positive selection, P), in line N50 for low performance (negative selection, N), and in line R, selection was random. Similar to lines P50 and N50, two additional lines (P25 and N25) were created by randomly sampling and mating 50 males and 300 females from line R in generation 25, again without replacement. Within each of these lines, mating continued for 25 generations with positive (P25) or negative (N25) selection. Finally, another two lines (P10 and N10) were created by randomly sampling and mating 50 males and 300 females from line R in generation 40. Within these lines, mating continued for 10 generations with positive (P10) or negative (N10) selection. Litter size was kept constant at 10 offspring (5 male and 5 female) in each of the breeding lines, and mating of selected parents was always at random. The average ${N}_{e}$ within the breeding lines was around ~ 115, which was calculated as $1/(2\Delta F)$, where $\Delta F$ is the inbreeding rate estimated using pedigree [19]. The simulated lines resemble real livestock breeding lines that are under selection, with a rate of inbreeding of about 0.5%.

#### Genome

The genome consisted of 10 chromosomes of 1 Morgan each. Each chromosome had 5000 randomly positioned bi-allelic loci. In the first historical generation, allele frequencies of these loci were sampled from a uniform distribution. During the historical generations, the mutation rate was 5.0 $\times$ 10^–5^, while there was no mutation after the historical generations. In generation 0 (i.e., after the last historical generation), the distribution of allele frequencies was U-shaped, and we randomly selected 1000 loci from those that segregated to become QTL. We did not simulate markers, because our interest was in the true value of ${r}_{pc}$, not in its estimation.

#### Functional genetic effects

The additive effect ($\mathrm{a}$) of each of the 1000 QTL was sampled from a normal distribution with mean 0 and variance 1. The size of non-additive effects at QTL was assumed to depend on the size of additive effects at these QTL. To achieve this, independently sampled dominance and epistatic coefficients for a QTL were scaled by the already sampled additive effects. Dominance coefficients ($\updelta$) were sampled from a normal distribution with a mean of 0.2 and a standard deviation of 0.3, following empirical observations by Bennewitz and Meuwissen [29] and Sun and Mumm [30]. Dominance effects ($\mathrm{d}$) were then computed by element-wise multiplication of $\updelta$ and $|\mathrm{a}|$. Epistatic interactions limited to pairs of QTL and each QTL had an epistatic interaction with five randomly sampled QTL. Epistatic interactions between pairs of QTL followed either the additive-by-additive (E_AA_) configuration, or the complementary (E_C_) configuration (Fig. 1), depending on the scenario. Epistatic coefficients ($\upgamma$) were sampled from a normal distribution with a mean of 0, because epistatic effects are likely non-directional [31]. The standard deviation of epistatic effects was set such that the total functional epistatic variance per QTL was comparable to the total functional dominance variance per QTL in scenario D. The total functional dominance variance at a QTL is equal to the squared mean dominance coefficient, plus the variance of dominance coefficients. Because each QTL was involved in five epistatic interactions but had only one dominance effect, the standard deviation of epistatic coefficients was set to $\sqrt{({0.2}^{2}+{0.3}^{2})/5}\approx 0.16$. Epistatic effects ($\upepsilon$) were computed as ${\gamma }_{kl}\sqrt{|{a}_{k}{a}_{l}|}$ for all pairwise interactions between QTL $k$ and $l$.

### Average effects and additive genetic values

For a single locus, the average effect for PB performance in line 1 (${\alpha }_{1}$) was computed from the functional genetic effects ($a$, $d,$ and $\epsilon$), and genotype frequencies in that line, as described in Duenk et al*.* [17], using the natural and orthogonal interactions (NOIA) model [32, 33]. The average effect for CB performance in line 1 when mated with line 2 (${\alpha }_{1(C)}$) was computed by the same procedure but with a small adjustment, as explained in Appendix 1.

Additive genetic values for PB performance in line 1 were computed as:

17 $${\mathbf{v}}_{1}={\mathbf{H}}_{1}{{\varvec{\upalpha}}}_{1}$$

where ${\mathbf{H}}_{1}$ is the $(n x m)$ QTL genotype matrix of animals in line 1 and ${{\varvec{\upalpha}}}_{1}$ is the $(m x 1)$ column vector of average effects for PB performance in line 1, where $n$ is the number of animals and $m$ is the number of QTL. Genotypes in ${\mathbf{H}}_{1}$ for individual $i$ at QTL $j$ were coded as in ${\mathbf{h}}_{i}$ (Eq. (2)), with elements:

18 $$h_{ij} = \left\{ {\begin{array}{*{20}c} {2 - 2p_{j} } \\ {1 - 2p_{j} } \\ {0 - 2p_{j} } \\ \end{array} } \right.{\text{for genotypes}},\,\left\{ {\begin{array}{*{20}c} {FF} \\ {Ff} \\ {ff} \\ \end{array} } \right.,$$

where ${p}_{j}$ is the frequency of allele $F$ at QTL $j$ in line 1. Additive genetic values for CB performance of animals in line 1 (when mated to line 2) (${\mathbf{v}}_{C}$) were computed by replacing ${{\varvec{\upalpha}}}_{1}$ with ${{\varvec{\upalpha}}}_{1(C)}$ in Eq. (17).

### Parameters of interest

The true value of ${r}_{pc}$ in line 1 when it is mated to line 2 was computed as the correlation between additive genetic values for PB (${\mathbf{v}}_{1}$) and CB performance (${\mathbf{v}}_{C}$) of animals in line 1, i.e. we did not estimate ${r}_{pc}$ from the simulated data. Note that this ${r}_{pc}$ is not the same as the ${r}_{pc}$ in line 2 when it is mated to line 1, because differences in allele frequencies between lines 1 and 2 lead to differences in contributions of QTL to the (co)variance of additive genetic values. In addition, average effects for CB performance in line 1 can differ from those in line 2 (e.g. based on Eqs. (5) and (6) for genetic model D). Thus, we computed ${r}_{pc}$ for all $7\times \left(7-1\right)=42$ combinations of breeding lines. All simulations were replicated 20 times, resulting in $42\times 20=840$ realized ${r}_{pc}$ values for each scenario.

We compared each of the realized ${r}_{pc}$ values with the theoretical predictions of ${r}_{pc}$ under genetic models D (${r}_{pc}^{D}$, Eq. (13)) and E_AA_ (${r}_{pc}^{AA}$, Eq. (16)). We expected that ${r}_{pc}^{D}$ and ${r}_{pc}^{AA}$ would exactly predict ${r}_{pc}$ in scenarios D and E_AA_, respectively. For scenarios D + E_AA_ and E_C_, ${r}_{pc}$ could not be expressed in terms of genetic parameters in the parental lines (see Discussion), but, as argued above, we expected that ${r}_{pc}^{D}$ may represent a lower bound and ${r}_{pc}^{AA}$ an upper bound of realized ${r}_{pc}$. Thus, it may be possible to predict the realized ${r}_{pc}$ for these two scenarios with a multiple regression model with ${r}_{pc}^{D}$ and ${r}_{pc}^{AA}$ as covariates.

## Results

**Fig. 3:**
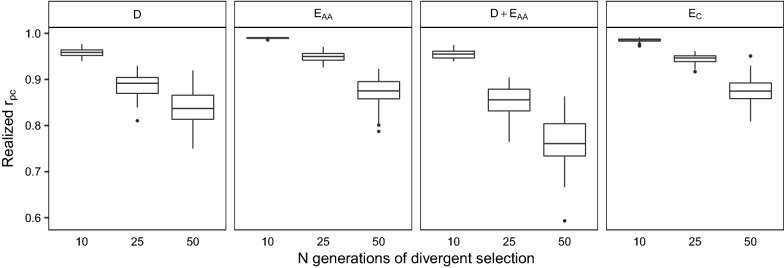
Realized ${r}_{pc}$ (y-axis) for crosses between lines that were divergently selected for 10 (P10-N10), 25 (P25-N25), or 50 generations of selection (P50-N50) (x-axis). Panels refer to the simulated genetic model

Figure 3 and Table S1 [see Additional file 1: Table S1] show the realized ${r}_{pc}$ for all crosses between lines that were divergently selected for either 10, 25, or 50 generations (i.e., crosses P10-N10, P25-N25, and P50-N50). For all scenarios (i.e. the four simulated genetic models), the realized ${r}_{pc}$ decreased with increasing generations of divergent selection, as expected. For each cross shown in Fig. 3, the lowest realized ${r}_{pc}$ was observed when both dominance and epistasis were simulated (scenario D + E_AA_), and the highest realized ${r}_{pc}$ was observed when only epistasis was simulated (scenarios E_AA_ and E_C_). Differences in ${r}_{pc}$ between scenarios were caused by differences in the genetic models between scenarios, rather than by differences in allele frequencies between lines, because mean differences in allele frequencies between lines were similar across scenarios (results not shown). This agrees with Duenk et al*.* [17].

**Fig. 4:**
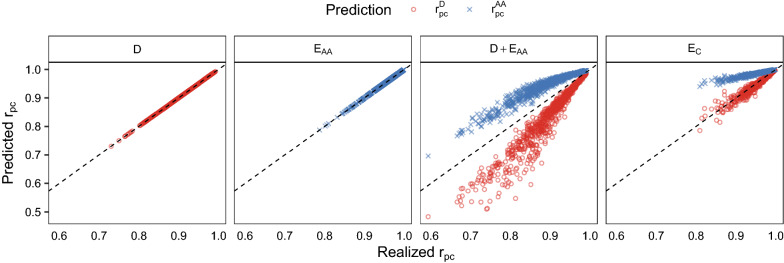
Predicted ${r}_{pc}$ (y-axis) for a genetic model with only dominance (${r}_{pc}^{D}$, red circles) and with only additive by additive epistasis (${r}_{pc}^{AA}$, blue crosses), plotted against the realized ${r}_{pc}$ (x-axis) in simulated scenarios. Panels refer to the simulated genetic model. The dashed lines show y = x, indicating where predictions are equal to the realized ${r}_{pc}$

Figure 4 shows the theoretical predictions of ${r}_{pc}$ from our expressions, plotted against the realized ${r}_{pc}$ from the simulations, for all replicates and for all combinations of parental lines within each replicate. For scenarios D and E_AA_, our expressions for ${r}_{pc}$ based on parameters in the purebred parental lines provided exact predictions of realized ${r}_{pc}$ (left two panels in Fig. 4). For scenarios D + E_AA_ and E_C_, our expressions for ${r}_{pc}$ were expected to provide upper (${r}_{pc}^{AA}$) and lower (${r}_{pc}^{D}$) bounds for realized ${r}_{pc}$. However, for scenario E_C_, realized ${r}_{pc}$ was lower than the lower bound (${r}_{pc}^{D}$) in 12% of the cases, for which realized ${r}_{pc}$ was about ~ 0.01 lower than ${r}_{pc}^{D}$. For both scenarios D + E_AA_ and E_C_, the gap between the predicted lower and upper bounds (i.e. the difference between ${r}_{pc}^{AA}$ and ${r}_{pc}^{D}$) increased with decreasing realized ${r}_{pc}$.

## Discussion

The aim of this study was to derive expressions for ${r}_{pc}$ for a purebred line when it is mated to another purebred line, based on genetic variances within, and the genetic covariance between the two parental lines. These expressions were derived for a genetic model with additive and dominance effects (model D), and for a genetic model with additive and additive-by-additive (A $\times$ A) epistatic effects (model E_AA_). The results showed that our expressions provide exact predictions of ${r}_{pc}$ for scenarios that were simulated based on models D and E_AA_, respectively. For scenarios with both dominance and A $\times$ A epistasis (D + E_AA_), and for models with complementary epistasis (E_C_), theoretical predictions could not be derived but the expressions for models D and E_AA_ provide approximate upper and lower bounds for ${r}_{pc}$, respectively. For the simulated D + E_AA_ scenario, the realized ${r}_{pc}$ always fell between these bounds, while for the simulated E_C_ scenario, the realized ${r}_{pc}$ was slightly lower than the lower bound in 12% of the cases.

The results of our simulations showed that the realized ${r}_{pc}$ decreased for all scenarios (i.e. the four genetic models simulated) when the number of generations of divergent selection between the parental lines increased. This was as expected because, with divergent selection, the difference in allele frequencies between parental lines increases over time, causing an increase in the differences between the average effects for PB and CB performance within each parental line [15, 16]. The realized ${r}_{pc}$ was lower for scenarios that involved dominance compared to scenarios that involved only epistasis. Across scenarios, the realized ${r}_{pc}$ ranged from 0.60 to 0.99, covering a large proportion of empirical estimates of ${r}_{pc}$ for livestock [3–9].

### Predicting ${{\varvec{r}}}_{{\varvec{p}}{\varvec{c}}}$ in practice

The expressions derived in this study suggest that the ${r}_{pc}$ can be predicted without CB information when certain variance components for the parental lines are known. Recent developments in genome-wide marker panels have made accurate estimation of the required variance components within and the covariances between distantly related lines feasible [18, 34, 35]. To validate our expressions as bounds for the ${r}_{pc}$ for a parental line, we attempted to apply them to empirical estimates reported in the literature. However, to our knowledge, only one study presents both an estimate of ${r}_{pc}$ based on PB and CB data, and of the corresponding genetic variances within, and of the covariances between the parental lines [36]. In that study, the estimate of ${r}_{pc}$ in the Yorkshire breed when mated to the Landrace breed was 0.67 with a standard error of 0.10. Based on the PB estimates presented in that paper, the predicted lower bound of ${r}_{pc}$ based on Eq. (13) was 0.30 and the predicted upper bound based on Eq. (16) was 0.84. These results suggest that it is unlikely that dominance is the only reason for the estimate of ${r}_{pc}$ in this study to be lower than 1, because the estimated ${r}_{pc}$ was higher than the lower bound. Thus, it is likely that epistasis or GxE is present.

Although our expressions appeared to predict lower and upper bounds for the above example, there are two important issues that arise when our expressions are applied to empirical data. First, ${r}_{pc}$ may be lower than the predicted lower bound given by Eq. (13), because our expressions do not account for G $\times$ E interactions. G $\times$ E interactions may be present in the study of Xiang et al*.* [36] because the PB and CB animals used in that study were housed in different environments. However, this implies that the results of our study can be used to evaluate the relative contributions of G $\times$ E and G $\times$ G to ${r}_{pc}$ by comparing estimates of ${r}_{pc}$ from PB and CB data with the predicted lower bound. For example, when the estimate of ${r}_{pc}$ is much lower than the predicted lower bound of ${r}_{pc}$, the contribution of G $\times$ E is likely large compared to the contribution of non-additive effects and differences in allele frequencies.

Second, the estimates of variance components obtained from empirical data are usually different from those used in Eqs. (13) and (16). In Xiang et al*.* [36] for example, a bivariate model was used to estimate genetic parameters within and between the two parental lines (say line 1 and 2). With such a model, the estimate of the genetic variance in line 2 refers to the variance in line 2 for the trait expressed in line 2 (i.e. ${\sigma }_{2}^{2}$). However, when the aim is to predict the bounds of ${r}_{pc}$ in line 1, we need the genetic variance in line 1 for the trait expressed in line 2 (i.e. ${\sigma }_{1\left(2\right)}^{2}$ in Eqs. (13) and (16)). Similarly, the covariance between lines 1 and 2 that is estimated from PB data from the two lines (${\sigma }_{\mathrm{1,2}}$) is not the same as the covariance between lines used in our expressions (${\sigma }_{\mathrm{1,1}(2)}$). Thus, as shown by our expressions, ${r}_{pc}$ based on genetic model D is different from to the genetic correlation between parental lines that is usually estimated in empirical studies (e.g., Xiang et al*.* [36]).

To predict ${r}_{pc}$ in line 1 when it is mated to line 2, there is no obvious way to directly estimate the variance (${\sigma }_{1\left(2\right)}^{2}$) and covariance (${\sigma }_{\mathrm{1,1}\left(2\right)}$) that are required for the expressions for ${r}_{pc}$, simply because the trait expressed in line 2 is not observed for individuals from line 1, but it may be possible to approximate them. One possible approach would be to estimate marker effects using genotype and phenotype data from line 2 and multiply them with the marker genotypes from line 1, resulting in genomic estimated breeding values (GEBV) for the animals in line 1 for the trait expressed in line 2. Parameter ${\sigma }_{1\left(2\right)}^{2}$ can then be approximated by the variance of these GEBV and parameter ${\sigma }_{\mathrm{1,1}\left(2\right)}$ by the covariance between these GEBV and the usual GEBV for PB performance in line 1. Although this approach appears straightforward, there are at least two issues that must be addressed. First, the LD between markers and QTL may be different in the parental lines, which leads to differences in estimated marker effects even when the QTL effects are not different [37]. Second, the estimated marker effects are subject to shrinkage to a degree that depends on the number of phenotypic records and the minor allele frequency of the marker. As a result, the variance of the estimated marker effects is smaller than the variance of the “true” marker effects. The effect of these issues on the predicted ${r}_{pc}$ requires further investigation.

### Three- and four-way crosses

The predictions of ${r}_{pc}$ presented here are valid for purebred parental lines that produce a two-way crossbred (i.e. from mating with one other purebred parental line). However in practice, commercial animals are usually three- or four-way crossbreds. The ${r}_{pc}$ for three- and four-way crosses under genetic models D and E_AA_ can also be expressed in terms of variances and covariances within and between parental lines. Derivation of these expressions is presented in Appendix 2. With genetic model D, ${r}_{pc}$ for the two dam lines that are involved in a three-way cross is equal to the ${r}_{pc}$ for two-way CB performance when the respective lines are mated directly to the sire line. This is because, alleles at a locus that are transmitted to a three-way cross from each of the dam lines will always pair with an allele from the sire line. Hence, following Eq. (13), ${r}_{pc}$ of a dam line for three-way CB performance is equal to the correlation between additive genetic values of the dams for the trait expressed in the dam line and in the sire line. For all other scenarios with three- or four-way crosses, the derivations of ${r}_{pc}$ result in expressions that differ from the expressions for two-way crosses (Appendix 2).

### Validity of predicted bounds of ${{\varvec{r}}}_{{\varvec{p}}{\varvec{c}}}$

We hypothesized that the predicted ${r}_{pc}$ under the dominance model (${r}_{pc}^{D}$) yields a lower bound for realized ${r}_{pc}$ because the difference between average effects for PB and CB performance is maximized with model D, since the average effect for CB performance at a QTL is a function of allele frequencies in the mated line. Our simulations showed that this lower bound was indeed correct for most cases, apart from a few replicates for the scenario of complementary epistasis (model E_C_). This is probably because, with model E_C_, the average effect for CB performance at a QTL involves a multiplication between the allele frequency of the same locus in the mated line, and the allele frequencies of the interacting loci in the cross. In contrast, for model D, the expression for the average effect for CB performance only involves the allele frequency of the same locus in the mated line (Eq. 6). As a consequence, with model E_C_, a difference in the allele frequency between parental lines at a QTL can result in differences in average effects between PB and CB performance at two QTL, instead of at only one QTL with model D.

## Conclusions

We derived expressions for ${r}_{pc}$ in purebred parental lines of two-, three-, and four-way crosses based on the genetic variances within and the genetic covariance between parental lines, noting that these variance components are not those that are typically estimated using empirical data. The expressions were derived for a genetic model with additive and dominance effects (model D), and for a model with additive and epistatic additive-by-additive effects (model E_AA_). Results showed that these expressions provide exact predictions of ${r}_{pc}$ for models D and E_AA_, and accurate upper and lower bounds of ${r}_{pc}$ for genetic models with both dominance and additive-by-additive epistatic effects (model D + E_AA_), or with complementary epistatic effects (model E_C_). This work lays the foundation for estimation of ${r}_{pc}$ based on information collected on the PB parental lines, without requiring CB information.

### Supplementary Information

**Additional file 1: Table S1.** Minimum, mean, and maximum values of realized r_pc for crosses between lines that were divergently selected for 10 (P10-N10), 25 (P25-N25), or 50 generations of selection (P50-N50).

## Data Availability

The data simulated in this study can be reproduced with the files in the following GitHub repository: https://git.wageningenur.nl/duenk002/predicting_rpc.

## Appendices

### Appendix 1

With non-additive genetic effects (i.e. dominance and epistasis), average effects of QTL are a function of genotype frequencies and functional genetic effects. The procedure to obtain average effects for PB performance from known genotype frequencies and functional additive, dominance, and epistatic effects, was described in Duenk et al*.* [17]. In short, the procedure involves applying the natural and orthogonal interactions (NOIA) model [32] for each epistatic interaction between two loci in a population with given allele frequencies, resulting in statistically orthogonal terms that contribute to the average effects of the two loci. Consider for example two loci, $k$ and $l$, that have an epistatic interaction between them. The functional epistatic values for each possible two-locus genotype can be partitioned into nine statistical genetic effects, using Eq. 2 in Duenk et al*.* [17]. For average effects for PB performance, the frequencies used in ${\mathbf{D}}_{kl}$,${\mathbf{W}}_{k}$ and ${\mathbf{W}}_{l}$ are those in the PB line. The procedure immediately leads to two terms (${\alpha }_{kl}^{k}$ and ${\alpha }_{kl}^{l}$) that contribute to the average effects of loci $k$ and $l$. For example, in a two-locus model where locus $k$ has an interaction with locus $l$ only, the average effect for PB performance of locus $k$ in line 1 is:

$${\alpha }_{1}^{k}={a}^{k}+\left(1-2{p}_{1}^{k}\right){d}^{k}+{\alpha }_{kl}^{k}$$

where ${a}^{k}$ is the functional additive effect of locus $k$, ${p}_{1}^{k}$ is the allele frequency of locus $k$ in line 1, and ${d}^{k}$ is the functional dominance effect of locus $k$. For PB performance, the average effect at locus $k$ depends on genotype frequencies of $k$ in line 1, because alleles of locus $k$ transmitted to PB animals always pair with an allele from the same line origin. Furthermore, the average effect of $k$ depends on genotype frequencies of locus $l$ in line 1, because alleles of locus $k$ transmitted to PB animals will be expressed in the genetic background of line 1.

The same procedure can be used to obtain average effects for CB performance for line 1, by making a small modification. The alleles of locus $k$ transmitted to two-way crossbreds always pair with an allele from line 2 and they will be expressed in the genetic background of crossbreds. Hence, the average effect for CB performance of locus $k$ depends on genotype frequencies of $k$ in line 2, and on genotype frequencies of $l$ in the crossbreds. Thus, to obtain the average effect at locus $k$ for CB performance, ${\mathbf{W}}_{k}$ needs to be constructed using the genotype frequencies of $k$ in line 2, ${\mathbf{W}}_{l}$ needs to be constructed using the genotype frequencies of $l$ in crossbreds, and ${\mathbf{D}}_{kl}$ is a (9 × 9) diagonal matrix of two-locus genotype probabilities, constructed using genotype frequencies of $k$ in line 2 and frequencies of $l$ in crossbreds. Then, the average effect at locus $k$ for CB performance in line 1 is:

$${\alpha }_{1(C)}^{k}={a}^{k}+\left(1-2{p}_{2}^{k}\right){d}^{k}+{{\alpha }^{\mathrm{^{\prime}}}}_{kl}^{k}$$

where ${p}_{2}^{k}$ is the allele frequency of locus $k$ in line 2. For the same epistatic interaction, the procedure needs to be repeated for locus $l$, because the average effect at locus $l$ for CB performance depends on genotype frequencies of $l$ in line 2 and on genotype frequencies of $k$ in the crossbreds.

### Appendix 2

#### Dam line of a three-way crossbred

Under genetic model D, the average effect at locus F for three-way CB performance for line 2 (or 3) is:

$${\alpha }_{2\left(C\right)}^{D}=0.5(a+\left(1-2{p}_{1}\right)d)$$

where ${p}_{1}$ is the allele frequency of allele $F$ in the sire line (line 1). The value $0.5$ is because an allele from this dam line has a probability of $0.5$ to be transmitted to the final crossbred. Given the expressions for average effects for PB performance, the average effect for CB performance for line 2 is:

$${\alpha }_{2\left(C\right)}^{D}=0.5{\alpha }_{1}$$

The additive genetic variance for three-way CB performance for line 2 under model D is:

$$\begin{aligned}{\sigma }_{2(C)}^{2} &=var\left({\mathbf{h}}_{i}^{\mathrm{^{\prime}}}{{\varvec{\upalpha}}}_{2(C)}\right)=var\left(\sum_{j}{h}_{ij}0.5{\alpha }_{1j}\right)\\ &=E\left[\left(\sum_{j}{h}_{ij}0.5{\alpha }_{1j}\right)\left(\sum_{j}{h}_{ij}0.5{\alpha }_{1j}\right)\right]\\ &=\sum_{j}E\left({h}_{ij}{h}_{ij}\right){\left(0.5{\alpha }_{1j}\right)}^{2}\\ &= 0.25\sum_{j}2{p}_{2j}\left(1-{p}_{2j}\right){\alpha }_{1j}^{2}=0.25{\sigma }_{2(1)}^{2}\end{aligned}$$

The additive genetic covariance between PB and three-way CB performance for line 2 under model D is:

$$\begin{aligned}{\sigma }_{\mathrm{2,2}\left(C\right)}&=cov\left({\mathbf{h}}_{i}^{\mathrm{^{\prime}}}{{\varvec{\upalpha}}}_{2},{\mathbf{h}}_{i}^{\mathrm{^{\prime}}}{{\varvec{\upalpha}}}_{2\left(C\right)}\right)\\ &=cov\left(\sum_{j}{h}_{ij}{\alpha }_{2j},\sum {h}_{ij}0.5{\alpha }_{1j}\right)\\ &=E\left[\left(\sum_{j}{h}_{ij}{\alpha }_{2j}\right)\left(\sum_{j}{h}_{ij}0.5{\alpha }_{1j}\right)\right] \\ &=\sum_{j}E\left({h}_{ij}{h}_{ij}\right)0.5{\alpha }_{2j}{\alpha }_{1j}\\ &=0.5\sum_{j}2{p}_{2j}\left(1-{p}_{2j}\right){\alpha }_{2j}{\alpha }_{1j}=0.5{\sigma }_{\mathrm{2,2}(1)}\end{aligned}$$

Hence, ${r}_{pc}$ for line 2 with genetic model D is equal to:

$${r}_{pc}^{D}=\frac{0.5{\sigma }_{\mathrm{2,2}(1)}}{{\sigma }_{2}\sqrt{0.25{\sigma }_{2(1)}^{2}}}=\frac{{\sigma }_{\mathrm{2,2}(1)}}{{\sigma }_{2}{\sigma }_{2(1)}}$$

This result shows that the ${r}_{pc}$ for a dam line of a three-way cross is equal to the genetic correlation between the traits for lines 1 and 2, as expressed in line 2. This result is similar to the expression of ${r}_{pc}$ in a sire line of a two-way cross.

Under genetic model E_AA_, the average effect at locus F for three-way CB performance for dam line 2 (or 3) is:

$${\alpha }_{2\left(C\right)}^{AA}=a-\left(1-2{p}_{C}^{G}\right)\epsilon$$

where ${p}_{C}^{G}$ is the allele frequency of allele $G$ in cross 1(23). Given the expressions for average effects for PB performance in lines 1, 2, and 3, and using ${p}_{C}^{G}=0.5{p}_{1}^{G}+0.25{p}_{2}^{G}+0.25{p}_{3}^{G}$, the average effect for three-way CB performance for line 2 under genetic model E_AA_ can be written as:

$${\alpha }_{2\left(C\right)}^{AA}=0.5{\alpha }_{1}+0.25{\alpha }_{2}+0.25{\alpha }_{3}$$

The additive genetic variance for three-way CB performance for line 2 with genetic model E_AA_ is:

$$\begin{aligned}{\sigma }_{2\left(C\right)}^{2}&=var\left({\mathbf{h}}_{i}^{\mathrm{^{\prime}}}{{\varvec{\upalpha}}}_{2\left(C\right)}\right)\\&=var\left(\sum_{j}{h}_{ij}\left(0.5{\alpha }_{1j}+0.25{\alpha }_{2j}+0.25{\alpha }_{3j}\right)\right)\\&=E\left[\left(\sum_{j}{h}_{ij}\left(0.5{\alpha }_{1j}+0.25{\alpha }_{2j}+0.25{\alpha }_{3j}\right)\right)\right.\\&\quad\left.\left(\sum_{j}{h}_{ij}\left(0.5{\alpha }_{1j}+0.25{\alpha }_{2j}+0.25{\alpha }_{3j}\right)\right)\right]\\&=\sum_{j}E\left({h}_{ij}{h}_{ij}\right){\left(0.5{\alpha }_{1j}+0.25{\alpha }_{2j}+0.25{\alpha }_{3j}\right)}^{2}\\&=0.25\sum_{j}2{p}_{2j}\left(1-{p}_{2j}\right)\left({\alpha }_{1j}^{2}+0.25{\alpha }_{2j}^{2}\right.\\&\left.+0.25{\alpha }_{3j}^{2}+{\alpha }_{1j}{\alpha }_{2j}+{\alpha }_{1j}{\alpha }_{3j}+ 2*0.25{\alpha }_{2j}{\alpha }_{3j}\right)\\&=0.25\left({\sigma }_{2(1)}^{2}+0.25{\sigma }_{2}^{2}+ 0.25{\sigma }_{2(3)}^{2}\right. \\ & \left.\quad+{\sigma }_{\mathrm{2,2}\left(1\right)}+{\sigma }_{2(1),2\left(3\right)}+0.5{\sigma }_{\mathrm{2,2}\left(3\right)}\right)\end{aligned}$$

Note that ${\sigma }_{2(1),2\left(3\right)}$ is the additive genetic covariance for individuals from line 2, between the trait expressed in lines 1 and 3.

The additive genetic covariance between PB performance and three-way CB performance for line 2 is:

$$\begin{aligned}{\sigma }_{\mathrm{2,2}\left(C\right)}&=cov\left({\mathbf{h}}_{i}^{\mathrm{^{\prime}}}{{\varvec{\upalpha}}}_{2},{\mathbf{h}}_{i}^{\mathrm{^{\prime}}}{{\varvec{\upalpha}}}_{2\left(C\right)}\right)\\&=cov\left(\sum_{j}{h}_{ij}{\alpha }_{2j},\sum {h}_{ij}\left(0.5{\alpha }_{1j}^{AA}+0.25{\alpha }_{2j}^{AA}+0.25{\alpha }_{3j}^{AA}\right)\right)\\&=E\left[\left(\sum_{j}{h}_{ij}{\alpha }_{2j}\right)\left(\sum_{j}{h}_{ij}\left(0.5{\alpha }_{1j}^{AA}+0.25{\alpha }_{2j}^{AA}+0.25{\alpha }_{3j}^{AA}\right)\right)\right]\\&=\sum_{j}E\left({h}_{ij}{h}_{ij}\right)0.5{\alpha }_{2j}\left(0.5{\alpha }_{1j}^{AA}+0.25{\alpha }_{2j}^{AA}+0.25{\alpha }_{3j}^{AA}\right)\\&=0.5\sum_{j}2{p}_{2j}\left(1-{p}_{2j}\right){\alpha }_{2j}\left({\alpha }_{1j}^{AA}+0.5{\alpha }_{2j}^{AA}+0.5{\alpha }_{3j}^{AA}\right)\\&=0.5\left({\sigma }_{\mathrm{2,2}\left(1\right)}+0.5{\sigma }_{2}^{2}+0.5{\sigma }_{\mathrm{2,2}\left(3\right)}\right)\end{aligned}$$

Hence, the ${r}_{pc}$ for line 2 under genetic model E_AA_ is:

$$\begin{aligned}{r}_{pc}^{AA}&=\frac{0.5\left({\sigma }_{\mathrm{2,2}\left(1\right)}+0.5{\sigma }_{2}^{2}+0.5{\sigma }_{\mathrm{2,2}\left(3\right)}\right)}{{\sigma }_{2}\sqrt{0.25\left({\sigma }_{2(1)}^{2}+0.25{\sigma }_{2}^{2}+0.25{\sigma }_{2(3)}^{2}+{\sigma }_{\mathrm{2,2}\left(1\right)}+{\sigma }_{2(1),2\left(3\right)}+0.5{\sigma }_{\mathrm{2,2}\left(3\right)}\right)}}\\ &=\frac{{\sigma }_{\mathrm{2,2}\left(1\right)}+0.5{\sigma }_{2}^{2}+0.5{\sigma }_{\mathrm{2,2}\left(3\right)}}{{\sigma }_{2}\sqrt{\left({\sigma }_{2(1)}^{2}+0.25{\sigma }_{2}^{2}+0.25{\sigma }_{2(3)}^{2}+{\sigma }_{\mathrm{2,2}\left(1\right)}+{\sigma }_{2(1),2\left(3\right)}+0.5{\sigma }_{\mathrm{2,2}\left(3\right)}\right)}}\end{aligned}$$

#### Sire line of a 3-way crossbred

Under genetic model D, the average effect at locus F for three-way CB performance for line 1 is:

$${\alpha }_{1\left(C\right)}^{D}=a+\left(1-2{p}_{23}\right)d$$

where ${p}_{23}$ is the allele frequency of allele $F$ in cross 23. Given the expressions for average effects for PB performance, and using ${p}_{23}=({p}_{2}+{p}_{3})/2$, the average effect for CB performance for line 1 is:

$${\alpha }_{1\left(C\right)}^{D}=0.5\left({\alpha }_{2}+{\alpha }_{3}\right)$$

The additive genetic variance for three-way CB performance for line 1 under model D is:

$$\begin{aligned}{\sigma }_{1\left(C\right)}^{2} &=var\left({\mathbf{h}}_{i}^{\mathrm{^{\prime}}}{{\varvec{\upalpha}}}_{1\left(C\right)}\right)=var\left(\sum_{j}{h}_{ij}0.5\left({\alpha }_{2j}+{\alpha }_{3j}\right)\right)\\ &=E\left[\left(\sum_{j}{h}_{ij}0.5\left({\alpha }_{2j}+{\alpha }_{3j}\right)\right)\left(\sum_{j}{h}_{ij}0.5\left({\alpha }_{2j}+{\alpha }_{3j}\right)\right)\right] \\ &=\sum_{j}E\left({h}_{ij}{h}_{ij}\right){\left(0.5\left({\alpha }_{2j}+{\alpha }_{3j}\right)\right)}^{2}\\ &= 0.25\sum_{j}2{p}_{2j}\left(1-{p}_{2j}\right)\left({\alpha }_{2j}{\alpha }_{2j}+{\alpha }_{3j}{\alpha }_{3j}+2{\alpha }_{2j}{\alpha }_{3j}\right)\\ &=0.25({\sigma }_{1(2)}^{2}+{\sigma }_{1(3)}^{2}+ \, 2{\sigma }_{1(2),1(3)})\end{aligned}$$

The additive genetic covariance between PB and three-way CB performance for line 1 under model D is:

$$\begin{aligned}{\sigma }_{\mathrm{1,1}\left(C\right)}&=cov\left({\mathbf{h}}_{i}^{\mathrm{^{\prime}}}{{\varvec{\upalpha}}}_{1},{\mathbf{h}}_{i}^{\mathrm{^{\prime}}}{{\varvec{\upalpha}}}_{1\left(C\right)}\right)\\ &=cov\left(\sum_{j}{h}_{ij}{\alpha }_{1j},\sum_{j}{h}_{ij}0.5\left({\alpha }_{2j}+{\alpha }_{3j}\right)\right)\\ &=E\left[\left(\sum_{j}{h}_{ij}{\alpha }_{1j}\right)\left(\sum_{j}{h}_{ij}0.5\left({\alpha }_{2j}+{\alpha }_{3j}\right)\right)\right]\\ &=\sum_{j}E\left({h}_{ij}{h}_{ij}\right)\left(0.5{\alpha }_{1j}{\alpha }_{2j}+0.5{\alpha }_{1j}{\alpha }_{3j}\right)\\ &=0.5\sum_{j}2{p}_{1j}\left(1-{p}_{1j}\right)\left({\alpha }_{1j}{\alpha }_{2j}+{\alpha }_{1j}{\alpha }_{3j}\right)\\ &=0.5\left({\sigma }_{\mathrm{1,1}\left(2\right)}+{\sigma }_{\mathrm{1,1}\left(3\right)}\right)\end{aligned}$$

Hence, the ${r}_{pc}$ for a sire line of a three-way cross with genetic model D is equal to:

$$\begin{aligned}{r}_{pc}^{D}&=\frac{0.5\left({\sigma }_{\mathrm{1,1}\left(2\right)}+{\sigma }_{\mathrm{1,1}\left(3\right)}\right)}{{\sigma }_{1}\sqrt{0.25\left({\sigma }_{1\left(2\right)}^{2}+{\sigma }_{1\left(3\right)}^{2}+2{\sigma }_{1\left(2\right),1\left(3\right)}\right)}}\\ &=\frac{{\sigma }_{\mathrm{1,1}\left(2\right)}+{\sigma }_{\mathrm{1,1}\left(3\right)}}{{\sigma }_{1}\sqrt{\left({\sigma }_{1\left(2\right)}^{2}+{\sigma }_{1\left(3\right)}^{2}+2{\sigma }_{1\left(2\right),1\left(3\right)}\right)}}\end{aligned}$$

Under genetic model E_AA_, the average effect at locus F for three-way CB performance for line 1 is:

$${\alpha }_{1\left(C\right)}^{AA}=a-\left(1-2{p}_{C}^{G}\right)\epsilon$$

where ${p}_{C}^{G}$ is the allele frequency of allele $G$ in cross 1(23). Given the expressions for average effects for PB performance for lines 1, 2, and 3, and using ${p}_{C}^{G}=0.5{p}_{1}^{G}+0.25{p}_{2}^{G}+0.25{p}_{3}^{G}$, the average effect for three-way CB performance for line 1 under genetic model E_AA_ can be written as:

$${\alpha }_{1\left(C\right)}^{AA}=0.5{\alpha }_{1}+0.25{\alpha }_{2}+0.25{\alpha }_{3}$$

The additive genetic variance for three-way CB performance for line 1 under model E_AA_ is:

$$\begin{aligned}{\sigma }_{1\left(C\right)}^{2} &=var\left({\mathbf{h}}_{i}^{\mathrm{^{\prime}}}{{\varvec{\upalpha}}}_{1\left(C\right)}\right)\\ &=var\left(\sum_{j}{h}_{ij}\left(0.5{\alpha }_{1j}+0.25{\alpha }_{2j}+0.25{\alpha }_{3j}\right)\right)\\ &=E\left[\left(\sum_{j}{h}_{ij}\left(0.5{\alpha }_{1j}+0.25{\alpha }_{2j}+0.25{\alpha }_{3j}\right)\right)\right. \\ & \left.\quad\left(\sum_{j}{h}_{ij}\left(0.5{\alpha }_{1j}+0.25{\alpha }_{2j}+0.25{\alpha }_{3j}\right)\right)\right]\\ &=\sum_{j}E\left({h}_{ij}{h}_{ij}\right){\left(0.5{\alpha }_{1j}+0.25{\alpha }_{2j}+0.25{\alpha }_{3j}\right)}^{2}\\ &= \sum_{j}2{p}_{1j}\left(1-{p}_{1j}\right)\left(0.25{\alpha }_{1j}^{2}+{0.25}^{2}{\alpha }_{2j}^{2}+{0.25}^{2}{\alpha }_{3j}^{2}\right. \\ & \left.\quad+0.25{\alpha }_{1j}{\alpha }_{2j}+0.25{\alpha }_{1j}{\alpha }_{3j}+0.125{\alpha }_{2j}{\alpha }_{3j}\right) \\ &=0.25\sum_{j}2{p}_{1j}\left(1-{p}_{1j}\right)\left({\alpha }_{1j}^{2}+0.25{\alpha }_{2j}^{2}\right. \\ & \left.\quad+0.25{\alpha }_{3j}^{2}+{\alpha }_{1j}{\alpha }_{2j}+{\alpha }_{1j}{\alpha }_{3j}+0.5{\alpha }_{2j}{\alpha }_{3j}\right)\\ &=0.25\left({\sigma }_{1}^{2}+ \, 0.25{\sigma }_{1(2)}^{2}+ \, 0.25{\sigma }_{1(3)}^{2}\right. \\ & \left.\quad+{\sigma }_{\mathrm{1,1}\left(2\right)}+{\sigma }_{\mathrm{1,1}\left(3\right)}+ \, 0.5{\sigma }_{1(2),1\left(3\right)}\right)\end{aligned}$$

The additive genetic covariance between PB and three-way CB performance for line 1 under genetic model E_AA_ is:

$$\begin{aligned}{\sigma }_{\mathrm{1,1}\left(C\right)}&=cov\left({\mathbf{h}}_{i}^{^{\prime}}{{\varvec{\upalpha}}}_{1},{\mathbf{h}}_{i}^{^{\prime}}{{\varvec{\upalpha}}}_{1\left(C\right)}\right)\\ &=cov\left(\sum_{j}{h}_{ij}{\alpha }_{1j},\sum_{j}{h}_{ij}\left(0.5{\alpha }_{1j}+0.25{\alpha }_{2j}+0.25{\alpha }_{3j}\right)\right)\\ &=E\left[\left(\sum_{j}{h}_{ij}{\alpha }_{1j}\right)\left(\sum_{j}{h}_{ij}\left(0.5{\alpha }_{1j}+0.25{\alpha }_{2j}+0.25{\alpha }_{3j}\right)\right)\right] \\ &=\sum_{j}E\left({h}_{ij}{h}_{ij}\right)\left(0.5{\alpha }_{1j}^{2}+0.25{\alpha }_{1j}{\alpha }_{2j}+0.25{\alpha }_{1j}{\alpha }_{3j}\right)\\ &=0.5\sum_{j}2{p}_{1j}\left(1-{p}_{1j}\right)\left({\alpha }_{1j}^{2}+0.5{\alpha }_{1j}{\alpha }_{2j}+0.5{\alpha }_{1j}{\alpha }_{3j}\right)\\ &=0.5\left({\sigma }_{1}^{2}+0.5{\sigma }_{\mathrm{1,1}\left(2\right)}+0.5{\sigma }_{\mathrm{1,1}\left(3\right)}\right)\end{aligned}$$

Hence, the ${r}_{pc}$ for line 1 for three-way CB performance under genetic model E_AA_ is equal to:

$$\begin{aligned}{r}_{pc}^{AA}&=\frac{0.5\left({\sigma }_{1}^{2}+0.5{\sigma }_{\mathrm{1,1}\left(2\right)}+0.5{\sigma }_{\mathrm{1,1}\left(3\right)}\right)}{{\sigma }_{1}\sqrt{0.25\left({\sigma }_{1}^{2}+0.25{\sigma }_{1\left(2\right)}^{2}+0.25{\sigma }_{1\left(3\right)}^{2}+{\sigma }_{\mathrm{1,1}\left(2\right)}+{\sigma }_{\mathrm{1,1}\left(3\right)}+0.5{\sigma }_{1\left(2\right),1\left(3\right)}\right)}}\\ &=\frac{{\sigma }_{1}^{2}+0.5{\sigma }_{\mathrm{1,1}\left(2\right)}+0.5{\sigma }_{\mathrm{1,1}\left(3\right)}}{{\sigma }_{1}\sqrt{\left({\sigma }_{1}^{2}+0.25{\sigma }_{1(2)}^{2}+0.25{\sigma }_{1(3)}^{2}+{\sigma }_{\mathrm{1,1}\left(2\right)}+{\sigma }_{\mathrm{1,1}\left(3\right)}+0.5{\sigma }_{1(2),1\left(3\right)}\right)}}\end{aligned}$$

#### Four-way cross

Under genetic model D, the average effect at locus F for four-way CB performance for line 1 is:

$${\alpha }_{1\left(C\right)}^{D}=0.5(a+\left(1-2{p}_{34}\right)d)$$

where ${p}_{34}$ is the allele frequency of allele $F$ in cross 34. The $0.5$ is because an allele from this parental line has a probability of $0.5$ to be transmitted to the final crossbred. Given the expressions for average effects for PB performance, and using ${p}_{34}=({p}_{3}+{p}_{4})/2$, the average effect for CB performance for line 1 is:

$${\alpha }_{1\left(C\right)}^{D}=0.25\left({\alpha }_{3}+{\alpha }_{4}\right)$$

The additive genetic variance for four-way CB performance for line 1 under model D is:

$$\begin{aligned}{\sigma }_{1\left(C\right)}^{2} &=var\left({\mathbf{h}}_{i}^{\mathrm{^{\prime}}}{{\varvec{\upalpha}}}_{1\left(C\right)}\right)\\&=var\left(\sum_{j}{h}_{ij}0.25\left({\alpha }_{3j}+{\alpha }_{4j}\right)\right)\\&=E\left[\left(\sum_{j}{h}_{ij}0.25\left({\alpha }_{3j}+{\alpha }_{4j}\right)\right)\left(\sum_{j}{h}_{ij}0.25\left({\alpha }_{3j}+{\alpha }_{4j}\right)\right)\right]\\&=\sum_{j}E\left({h}_{ij}{h}_{ij}\right){\left(0.25\left({\alpha }_{3j}+{\alpha }_{4j}\right)\right)}^{2}\\&= \sum_{j}2{p}_{1j}\left(1-{p}_{1j}\right){0.25}^{2}\left({\alpha }_{3j}^{2}+{\alpha }_{4j}^{2}+2{\alpha }_{3j}{\alpha }_{4j}\right)\\&={0.25}^{2}\sum_{j}2{p}_{1j}\left(1-{p}_{1j}\right)\left({\alpha }_{3j}^{2}+{\alpha }_{4j}^{2}+2{\alpha }_{3j}{\alpha }_{4j}\right)\\&= {0.25}^{2}({\sigma }_{1(3)}^{2}+{\sigma }_{1(4)}^{2}+2{\sigma }_{1(3),1(4)})\end{aligned}$$

The additive genetic covariance between PB and four-way CB performance for line 1 under model D is:

$$\begin{aligned}{\sigma }_{\mathrm{1,1}\left(C\right)}&=cov\left({\mathbf{h}}_{i}^{\mathrm{^{\prime}}}{{\varvec{\upalpha}}}_{1},{\mathbf{h}}_{i}^{\mathrm{^{\prime}}}{{\varvec{\upalpha}}}_{1\left(C\right)}\right)\\ &=cov\left(\sum_{j}{h}_{ij}{\alpha }_{1j},\sum_{j}{h}_{ij}0.25\left({\alpha }_{3j}+{\alpha }_{4j}\right)\right)\\ &\quad E\left[\left(\sum_{j}{h}_{ij}{\alpha }_{1j}\right)\left(\sum_{j}{h}_{ij}0.25\left({\alpha }_{3j}+{\alpha }_{4j}\right)\right)\right] \\ &=\sum_{j}E\left({h}_{ij}{h}_{ij}\right){\alpha }_{1j}\left(0.25\left({\alpha }_{3j}+{\alpha }_{4j}\right)\right)\\ &= 0.25\sum_{j}2{p}_{1j}\left(1-{p}_{1j}\right)\left({\alpha }_{1j}{\alpha }_{3j}+{\alpha }_{1j}{\alpha }_{4j}\right)\\ &=0.25({\sigma }_{\mathrm{1,1}\left(3\right)}+{\sigma }_{\mathrm{1,1}\left(4\right)})\end{aligned}$$

Hence, the ${r}_{pc}$ for line 1 under genetic model D is equal to:

$${r}_{pc}^{D}=\frac{0.25\left({\sigma }_{\mathrm{1,1}\left(3\right)}+{\sigma }_{\mathrm{1,1}\left(4\right)}\right)}{{\sigma }_{1}\sqrt{{0.25}^{2}\left({\sigma }_{1\left(3\right)}^{2}+{\sigma }_{1\left(4\right)}^{2}+2{\sigma }_{1\left(3\right),1\left(4\right)}\right)}}=\frac{{\sigma }_{\mathrm{1,1}(3)}+{\sigma }_{\mathrm{1,1}(4)}}{{\sigma }_{1}\sqrt{({\sigma }_{1(3)}^{2}+{\sigma }_{1(4)}^{2}+2{\sigma }_{1(3),1(4)})}}$$

This shows that, under genetic model D, the ${r}_{pc}$ for four-way CB performance is similar to the ${r}_{pc}$ for three-way CB performance for a sire line.

Under genetic model E_AA_, the average effect at locus F for four-way CB performance for line 1 is:

$${\alpha }_{1\left(C\right)}^{AA}=a-\left(1-2{p}_{C}^{G}\right)\epsilon$$

where ${p}_{C}^{G}$ is the allele frequency of allele $G$ for cross (12)(34). Given the expressions for average effects for PB performance in lines 1, 2, 3, and 4, and using ${p}_{C}^{G}=0.25{p}_{1}^{G}+0.25{p}_{2}^{G}+0.25{p}_{3}^{G}+0.25{p}_{4}^{G}$, the average effect for four-way CB performance for line 1 under genetic model E_AA_ can be written as the average of average effects for PB performance:

$${\alpha }_{1\left(C\right)}^{AA}=0.25{\alpha }_{1}+0.25{\alpha }_{2}+0.25{\alpha }_{3}+0.25{\alpha }_{4}$$

The additive genetic variance for four-way CB performance for line 1 can be written as:

$$\begin{aligned}{\sigma }_{1\left(C\right)}^{2} &=var\left({\mathbf{h}}_{i}^{\mathrm{^{\prime}}}{{\varvec{\upalpha}}}_{1\left(C\right)}\right)\\ &=var\left(\sum_{j}{h}_{ij}\left(0.25{\alpha }_{1}+0.25{\alpha }_{2}+0.25{\alpha }_{3}+0.25{\alpha }_{4}\right)\right)\\ &=E\left[{\left(\sum_{j}{h}_{ij}\left(0.25{\alpha }_{1}+0.25{\alpha }_{2}+0.25{\alpha }_{3}+0.25{\alpha }_{4}\right)\right)}^{2}\right] \\ &=\sum_{j}E\left({h}_{ij}{h}_{ij}\right){\left(0.25{\alpha }_{1}+0.25{\alpha }_{2}+0.25{\alpha }_{3}+0.25{\alpha }_{4}\right)}^{2}\\ &={0.25}^{2} \sum_{j}2{p}_{1j}\left(1-{p}_{1j}\right)\left(\sum_{l=1}^{4}{\alpha }_{lj}^{2}+\sum_{l=1}^{4}\sum_{k\ne l}^{4}{\alpha }_{lj}{\alpha }_{kj}\right)\\ &= 0.25^{2}\left(\sum_{l=1}^{4}{\sigma }_{1\left(l\right)}^{2}+\sum_{l=1}^{4}\sum_{\begin{array}{c}k=1\\ k\ne l\end{array}}^{4}{\sigma }_{1\left(l\right),1\left(k\right)}\right)\end{aligned}$$

while defining that ${\sigma }_{1\left(1\right)}^{2}={\sigma }_{1}^{2}$, and that ${\sigma }_{1\left(1\right),1(2)}={\sigma }_{\mathrm{1,1}\left(2\right)}$. The above notation is the sum of all variances and covariances for individuals from line 1 for the traits expressed in the four parental lines.

The additive genetic covariance between PB and four-way CB performance for line 1 is:

$$\begin{aligned}{\sigma }_{\mathrm{1,1}\left(C\right)}&=cov\left({\mathbf{h}}_{i}^{\mathrm{^{\prime}}}{{\varvec{\upalpha}}}_{1},{\mathbf{h}}_{i}^{\mathrm{^{\prime}}}{{\varvec{\upalpha}}}_{1\left(C\right)}\right)\\ &=cov\left(\sum_{j}{h}_{ij}{\alpha }_{1j},\sum_{j}{h}_{ij}\left(0.25{\alpha }_{1}+0.25{\alpha }_{2}+0.25{\alpha }_{3}+0.25{\alpha }_{4}\right)\right)\\ &=E\left[\left(\sum_{j}{h}_{ij}{\alpha }_{1j}\right)\left(\sum_{j}{h}_{ij}\left(0.25{\alpha }_{1}+0.25{\alpha }_{2}+0.25{\alpha }_{3}+0.25{\alpha }_{4}\right)\right)\right] \\ &=0.25\sum_{j}E\left({h}_{ij}{h}_{ij}\right){\alpha }_{1j}\left({\alpha }_{1}+{\alpha }_{2}+{\alpha }_{3}+{\alpha }_{4}\right)\\ &=0.25\sum_{j}2{p}_{1j}\left(1-{p}_{1j}\right)\sum_{l=1}^{4}{\alpha }_{1j}{\alpha }_{lj}=0.25\sum_{l=1}^{4}{\sigma }_{\mathrm{1,1}\left(l\right)}\end{aligned}$$

while defining that ${\sigma }_{\mathrm{1,1}\left(1\right)}={\sigma }_{1}^{2}$.

Hence, the ${r}_{pc}$ for line 1 with genetic model E_AA_ is equal to:

$${r}_{pc}^{AA}=\frac{0.25\sum_{l=1}^{4}{\sigma }_{\mathrm{1,1}\left(l\right)}}{{\sigma }_{1}\sqrt{{0.25}^{2}\left(\sum_{l=1}^{4}{\sigma }_{1\left(l\right)}^{2}+\sum_{l=1}^{4}\sum_{\begin{array}{c}k=1\\ k\ne l\end{array}}^{4}{\sigma }_{1\left(l\right),1\left(k\right)}\right)}}=\frac{\sum_{l=1}^{4}{\sigma }_{\mathrm{1,1}(l)}}{{\sigma }_{1}\sqrt{\sum_{l=1}^{4}{\sigma }_{1\left(l\right)}^{2}+\sum_{l=1}^{4}\sum_{\begin{array}{c}k=1\\ k\ne l\end{array}}^{4}{\sigma }_{1\left(l\right),1\left(k\right)}}}$$
