# Supplementary material for: Predicting the purebred-crossbred genetic correlation from the genetic variance components in the parental lines
Source: Genet Sel Evol. 2021 Feb 4;53:10. doi: 10.1186/s12711-021-00601-w (PMC7860586; doi:10.1186/s12711-021-00601-w)
Supplement: Supplementary file 1 — Additional file 1: Table S1. Minimum, mean, and maximum values of realized r_pc for crosses between lines that were divergently selected for 10 (P10-N10), 25 (P25-N25), or 50 generations of selection (P50-N50). [file 12711_2021_601_MOESM1_ESM.docx]

*Table S 1* ***Minimum, mean, and maximum values of realized*** $\boldsymbol{r}_{\boldsymbol{pc}}$ ***for crosses between lines that were divergently selected for 10 (P10-N10), 25 (P25-N25), or 50 generations of selection (P50-N50).***

| scenario | N generations | min | mean | max |
| --- | --- | --- | --- | --- |
| D | 10 | 0.94 | 0.96 | 0.98 |
| D | 25 | 0.81 | 0.89 | 0.93 |
| D | 50 | 0.75 | 0.84 | 0.92 |
|  |  |  |  |  |
| E_AA_ | 10 | 0.99 | 0.99 | 0.99 |
| E_AA_ | 25 | 0.93 | 0.95 | 0.97 |
| E_AA_ | 50 | 0.79 | 0.87 | 0.92 |
|  |  |  |  |  |
| D + E_AA_ | 10 | 0.94 | 0.95 | 0.97 |
| D + E_AA_ | 25 | 0.76 | 0.85 | 0.90 |
| D + E_AA_ | 50 | 0.59 | 0.76 | 0.86 |
|  |  |  |  |  |
| E_C_ | 10 | 0.97 | 0.99 | 0.99 |
| E_C_ | 25 | 0.92 | 0.94 | 0.96 |
| E_C_ | 50 | 0.81 | 0.88 | 0.95 |
